# Impact of participatory training of smallholder pig farmers on knowledge, attitudes and practices regarding biosecurity for the control of African swine fever in Uganda

**DOI:** 10.1111/tbed.13587

**Published:** 2020-05-17

**Authors:** Michel Mainack Dione, Ian Dohoo, Nicholas Ndiwa, Jane Poole, Emily Ouma, Winfred Christine Amia, Barbara Wieland

**Affiliations:** ^1^ International Livestock Research Institute Ouagadougou Burkina Faso; ^2^ University of Prince Edward Islands Charlottetown PE Canada; ^3^ International Livestock Research Institute Nairobi Kenya; ^4^ International Livestock Research Institute Kampala Uganda; ^5^ International Livestock Research Institute Addis Ababa Ethiopia

**Keywords:** African swine fever, biosecurity, participatory training, pig, Uganda

## Abstract

We evaluated the impact of a participatory training of pig farmers on knowledge, attitude and practices (KAP) of biosecurity relating to ASF control in two districts of Uganda using a randomized control trial (RCT). A total of 830 pig farmers from 32 villages were included in the study, with 425 farmers receiving training, while 405 did not. An item response theory model was used to assess the impact of the training on farmer's KAP. Logistic regression models were used to assess the factors that affected knowledge gain and change in attitude and practices after training. Focus group discussions (FGD) were carried out with selected farmers from the treatment group at the end of the intervention, to share their experiences and discuss potential factors that could hinder adoption of biosecurity in their communities. Results of the regression analyses showed that there was a significant effect of biosecurity training (*p* = .038) on gain in knowledge after 12 months, but there were limited changes in farmer's attitude and practice at 12 and 28 months after training. Pig production domain (peri‐urban/urban production), group membership, gender (male) and education of the farmer positively influenced knowledge gain and attitude of farmers towards biosecurity. This paper provides empirical evidence on the impact of training intervention on biosecurity practices for disease prevention or control. In addition, it breaks down the components of the biosecurity practices and documents the specific challenges to its uptake by the farmers. It therefore relaxes the assumption of knowledge constraint as a barrier to uptake. The results clearly show that knowledge is not the binding constraint to uptake of the biosecurity interventions.

## 1 INTRODUCTION

African swine fever (ASF) is a highly contagious viral disease which affects domestic pigs and wild boars. It can cause 100% mortality when introduced into a naïve pig herd. The disease is endemic in Uganda (Atuhaire et al., 2013) and causes high socio‐economic losses to pig farmers, especially smallholders (Chenais et al., 2017). In addition, the national disease surveillance and reporting systems are weak in developing countries, leading to under‐reporting of ASF outbreaks (Dione et al., 2014, 2015).There is currently no effective treatment or vaccine available, and therefore, implementation of strict farm biosecurity measures is the main tool for prevention and control of ASF. Our study was informed by previous research where participatory approaches through focus group discussions and key informant interviews were used to assess the constraints and opportunities for farmers and other actors along the smallholder pig value chains (Ouma et al., 2015). Research focused on pig health constraints (Dione et al., 2014) and biosecurity along the pig value chains (Dione, Ochago, Ouma, Lule, & Birungi, 2016; Dione, Ouma, Opio, Kawuma, & Pezo, 2016). According to the pig farmers, the lack of knowledge about best practices in biosecurity was a major challenge to the control of ASF and other pig diseases in Uganda. They thus recommended actions such as farmer trainings and experiential learning as an avenue to minimize the knowledge gaps. Adoption of biosecurity measures requires that farmers have knowledge of a range of infectious diseases and have the capacity to adopt biosecurity protocols, so they can easily apply them whenever necessary. Knowledge on biosecurity is a key driver in influencing behaviour (Cui & Liu, 2016; Young, Evans‐Kocinski, Bush, & Windsor, 2015). Recent studies in the smallholder pig systems in Uganda revealed that lack of knowledge of farmers on biosecurity, coupled with adoption of high‐risk practices and non‐compliance with regulations, has contributed to the persistence of ASF (Dione et al., 2015; Nantima et al., 2016). Hence, to reduce the burden of ASF in Uganda, farmer's capacity to apply biosecurity measures needs to be enhanced. We hypothesize that farmers would adopt and implement biosecurity protocols, if they have a sound understanding of the transmission patterns of ASF and if they know best practices in biosecurity and understand the promising effects of the approaches suggested. The challenge is to identify the best procedures to increase this understanding. Participatory training is an interactive learning process enabling individuals and communities to develop skills, knowledge and attitudes, and to share lessons learnt (Wilde & Vainio‐Mattila, 1995). During participatory training, participants are encouraged to explore and discover for themselves. Knowledge obtained this way is more easily internalized and put into practice (Komáromi, Kiss, & Pálinkás, 2010). It is centred on the farmers and developed according to their needs. Farmers understand the importance of the problem in relation to their activity and to what extend it can affect their livelihood if not addressed. They feel ownership of the whole process, in this way; they are participating in solving their own problems (Wilde & Vainio‐Mattila, 1995). This study investigated the effect of participatory training of smallholder pig farmers on KAP of good biosecurity practices and the readiness of farmers to implement these practices to reduce productive losses due to ASF.

## 2 MATERIALS AND METHODS

### 2.1 Site selection

**Figure 1:**
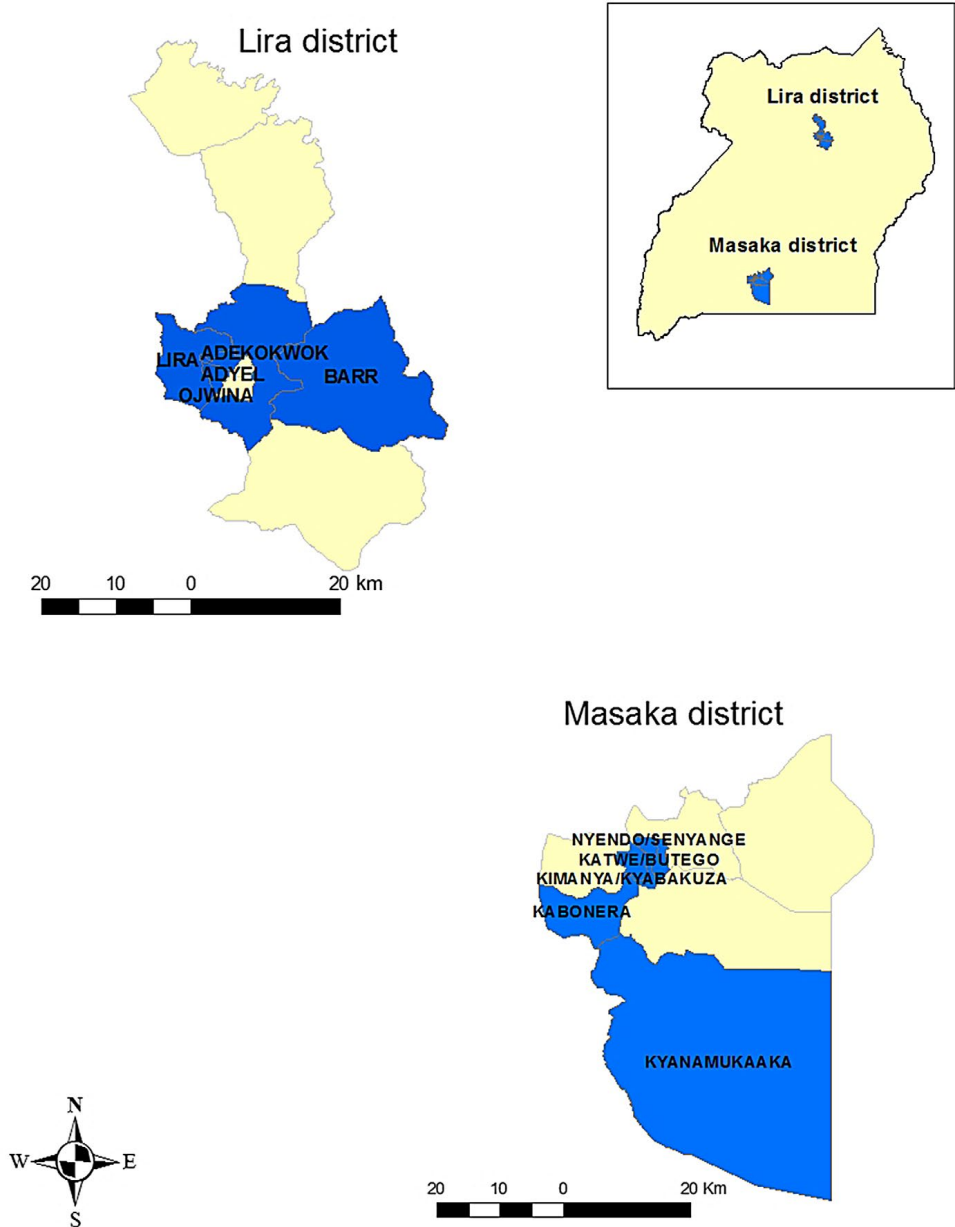
Map of Uganda with the study areas

The study was carried out in Masaka and Lira districts of Uganda. Masaka is located in the central region and has the highest pig population density in the country (>50 heads/km^2^) (UBOS, 2009), while Lira is located in the northern part of the country with a lower pig population density (Figure 1). These districts were part of the ‘Smallholder Pig Value Chains Development Project’ (SPVCD) in Uganda which is a research for development programme running since 2011 to improve pig production in the country (Ouma & Kawuma, 2014). In each district, villages with high pig population density were identified during a census*.* Areas with the highest ASF outbreaks, based on records from respective district veterinary offices, were considered as a proxy for high pig population density. The top 16 villages of each district were then selected and enrolled in the study, making 32 villages in total. Villages were randomly and equally allocated to treatment and control groups.

### 2.2 Sample size calculation, participant selection and randomization

The target population consisted of pig keepers at the time of the study. We assumed that 35% of farmers had adequate knowledge at the start based on expert opinion. We expected to increase knowledge to 65% of farmers after training. An intracluster correlation coefficient (ICC) of 0.38 was used for the sample size calculation. The ICC was set high because interaction and exchange of farmers in our study area were high. The required sample size was 27 pig‐keeping households per village with 80% power at 5% level of significance. Finally, we increased the number of households per villages to 30 to account for possible farmer's non‐compliance. In total, we included 480 households in the treatment group and 480 households in the control group. The level of randomization was the village, but the outcome of measurement was the individual farmer. The training was applied at the village level, meaning all pig keepers in selected villages in the treatment group were invited for the training in biosecurity. All pig‐keeping households in the selected villages were registered. In each village, 30 farmers who consented to participate in the study were randomly selected for the KAP assessments at baseline and endline.

### 2.3 Participatory training

Prior to the study, a training manual was developed by the project team (Nantima, Dione, Brandes‐van Dorresteijn, Kawuma, & Smith, 2015). The content of the training was focused on transmission and spread of ASF as well as measures for its control and prevention. Emphasis was put on key biosecurity measures that could make a difference in the control of ASF such as pig confinement, farm visit restriction, management of sick animals, disposal of dead animals, processing of swill, disinfection and outbreak reporting. Another manual was developed on the delivery of the content of the course (Dione, Ochago, Lule, & Mayega, 2018). Finally, a one‐page poster with illustration of key messages on ASF and biosecurity was developed and translated into local languages for distribution to farmers (Kramer, Dione, & Wieland, 2015). The manual's content and the training approach were, respectively, validated and tested with farmers and district veterinary extension personnel. The training of farmers was administered by extension staff from respective district veterinary offices to all consenting pig farmers in the villages that belonged to the treatment group. The extension officers were trained by the project team on how to administer the training. Farmers were split into groups of 20 to 30 people per training session which lasted about four hours. A coffee break and lunch breaks were organized for all participants. Each group was led by a facilitator and a note‐taker who recorded key issues that were discussed during the sessions. The training approach followed Dione et al. (2018) procedures. It was focused on ASF and application of biosecurity. The course was made of five sessions: ASF causes, symptoms and transmission (one hr); biosecurity measures at farm level (one hour); proper control of pig movements and reporting (30 min); on‐farm practical demonstration of biosecurity measures (one hr); and training evaluation (30 min). Since the target of the training was to improve farmer's knowledge of biosecurity, we focused more on knowledge and skill‐based lessons. Several delivery methods were used during the training including plenary, brainstorming, small group discussion, story storytelling awareness and practical demonstration of cleaning and disinfecting a pigsty, construction of a footbath, hand washing and disinfection, use of protective wears, swill processing and disposal of dead pigs. Various tools/ aids were used to relay the messages including photographs (of diseased pigs), posters, film clips and drawings. Farmers who faced ASF outbreaks could share their experiences with others to stimulate discussions among participants who then reflected on the strength and weaknesses of the biosecurity measures they had applied to control the disease. At the end of the fourth session, farmers were asked to evaluate the training in relation to its relevance to their expectations. They were also asked to provide recommendations for sustainable control of ASF and other pig diseases in their communities. Refresher training sessions were carried out 3 months after the first training with farmers who participated in the baseline KAP survey only. During the refresher training, farmers received a one‐pager poster with illustrations of key facts on ASF and biosecurity measures in their local languages.

### 2.4 Baseline KAP survey on biosecurity by farmers

The KAP questionnaire was a mixture of closed and Likert scale‐type questions on biosecurity (Table S1). The survey was carried out in April 2015. Enumerators were selected from the district extension personnel and were trained on the survey tools which they administered to farmers in local languages. They interviewed farmers at their homes after booking for an appointment facilitated by the area veterinarian. In order to minimize the risk of contaminating farms due to their movements, the enumerators observed strict biosecurity measures during their visits to homes. They were provided with disposable laboratory coats, gumboots and disinfectants to be used from farm to farm. Self‐reporting was verified by observation of the farm by the interviewer whenever possible. To better explain the Likert scale‐type questions to farmers, visual illustrations were created on the floor using pieces of manila papers for responses to be written in local languages.

### 2.5 Endline KAP survey on biosecurity after 12 and 28 months

A second KAP survey was carried out at 12 months (May 2016) on the same farmers in both the treatment and control groups, and a third KAP survey was carried out at 28 months (September 2017) involving farmers who belonged to the treatment group only. At the end of the study and for ethical reasons, all farmers from the control group were trained using the same biosecurity training material that was used for the treatment group.

### 2.6 Focus group discussion

Focus discussions were carried out as learning process at the end of the intervention. It was a short discussion with few farmers to collect feedback on what farmers considered as challenges in the implementation of what they have learnt from the training. The same people who implemented the training were used to lead the discussion with farmers in local languages under the supervision of the researchers. Four FGDs made of men and women with an average of eight participants per group were carried out in each district. Participants were selected because they were actively involved in the training and they were willing to share their experience. The notes taken during the discussion were combined, summarized and then presented in the tables under some specific themes that were defined by the authors.

### 2.7 Ethical consideration

A study information sheet describing the aim of the project was explained to all participants before the study either in English or in local languages. Farmers who were willing to participate in the study were asked to sign a consent form. Farmers in the control groups were trained and received awareness information on ASF control and biosecurity at the end of the intervention. This study was approved by the Uganda National Committee for Scientific Technology with approval reference number A508 and by the Institutional Ethical Review Committee of College of Veterinary Medicine, Animal Resources and Biosecurity of Makerere University Uganda with approval reference SBLS.MD.2015.

### 2.8 Data management and analysis

#### 2.8.1 Biosecurity knowledge

The data collected were entered into a CSPro database with inbuilt data validation features and then exported to STATA 15 for data management and analysis. Collected data on biosecurity knowledge and attitude using a five‐point Likert scale were recoded such that the correct or desirable responses (when the answers were in agreement with the protocol's recommendation) received a higher score up to five (5), while the least desired or wrong responses (when the answers were not in agreement with the protocol's recommendation) were recoded as one (1). The assessment of the responses was carried out by a team of veterinarians and epidemiologists who are well knowledgeable about biosecurity practices. The five‐point Likert scale for biosecurity knowledge was then converted into binary format by recoding 1 and 2 into zero (0) indicating wrong response, while 4 and 5 were recoded as one (1) indicating correct response. Scores of 3 were ignored because it was assumed to indicate neutral/undecided/not sure. The binary responses were then included in an item response theory (IRT) model which has procedures for analysing and obtaining information about the respondents, the questions asked (items) and the latent variable of interest, in this case knowledge of farmers on ASF. IRT models are used to summarize responses to a group of questions into an overall estimate of the factor of interest (e.g. knowledge of ASF). A fundamental assumption of IRT is that there is a unidimensional latent variable representing that factor of interest. This was evaluated by applying factor analysis to the set of questions and deleting items which did not load primarily on the first factor. Subsequently, a two‐parameter logistic (2PL) IRT model was fit to the retained items (questions) and items with very low discriminatory power were removed. The remaining items (*n* = 8) were used to create item characteristic curves (ICC) and item information functions. In the 2PL, the respondent's choice of the correct or wrong answer is dependent on the respondent's ability (knowledge) level, the item difficulty and its discrimination. Item discrimination is the degree to which an item differentiates individuals with high knowledge level from individuals with low knowledge level; while item's difficulty reflects the knowledge, level required for a respondent to have a 50% chance of answering the question (item) correctly. The individual respondent's overall knowledge (latent trait – designated theta) was estimated using an empirical Bayes estimator. Change in knowledge (after training – before training), as measured by theta, was analysed using a mixed‐effects linear model. Village was included as a random effect, while district and gender were included as fixed effects (potential confounders). Treatment group (trained versus control) was included as a fixed effect as was the before training knowledge latent variable (theta).

#### 2.8.2 Biosecurity attitude

Analysis of attitude variables was carried out one at a time because it was not possible to identify a single underlying trait that was being captured by the attitude variables. Random‐effects logistic regression (with village as a random effect and district and treatment forced in as fixed effects) was used to assess factors that influenced farmer's attitude towards allowing traders and veterinarians to enter their farms during outbreaks and decision to sell the pigs during outbreaks.

#### 2.8.3 Biosecurity practice

Similar to knowledge and attitude, biosecurity practice question responses were categorized into desirable and non‐desirable practices. The good/recommended practices were assigned a value of one (1) and the bad practices assigned a value of zero (0). IRT was considered not applicable for the biosecurity practice questions because these were direct questions not measuring any underlying variable and therefore each question was analysed separately. Random‐effects logistic regression (with village as a random effect and district and treatment forced in as fixed effects) was used to evaluate the effects of key factors on adoption of biosecurity practices.

### 2.9 Focus group discussion

Field note takers captured group discussions in the local languages and then translated them into English in the form of a report. Analysis of data involved process tracking, extraction and linking information on the key aspects of the study to pre‐identified themes. A list on factors affecting implementation of biosecurity measures and related explanations was established.

## 3 RESULTS

### 3.1 Demographic characteristics of the participants

A total of 830 pig‐keeping households participated in the pre‐ and post‐training surveys, 405 from the control group (49%) and 425 in the treatment group (51%). Seventy‐five farmers in the treatment group and 55 farmers in the control group were not reachable during the surveys. Although most of the sampled households (76%) were male‐headed, 58% of the respondents were female (Table S2).

**Figure 2:**
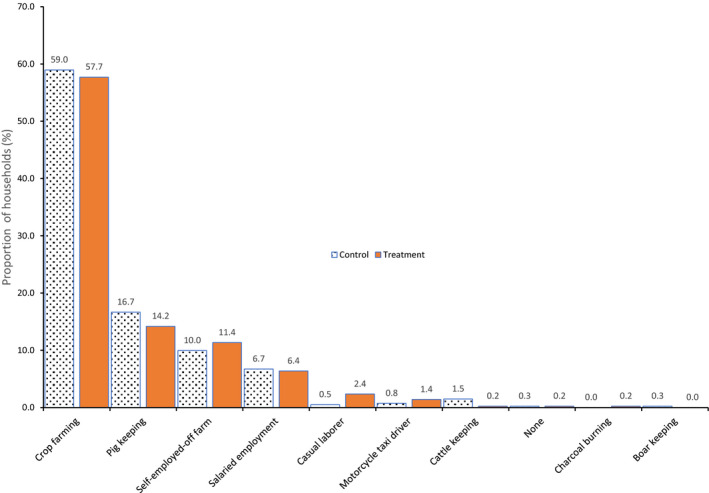
Main household income sources

The average age for respondents in both the treatment and control groups was 47 years with standard deviation of 14. On average, participants in the treatment group reported that they had been in the pig business for six years, while those in the control group have been in the business for four years. Crop and pig farming were the main sources of income for households in both groups (Figure 2).

### 3.2 Impact of training on farmer's knowledge

Eighty‐eight per cent (88%) respondents wrongly said that processing meat from dead pigs with ASF is not safe for human consumption (f59) and 65% said that their pigs can get sick when veterinarians without protective biosecurity measures get close to the pigs (f49) implying that these items were the most difficult for farmers to answer correctly. All other questions including all the questions included in the IRT model were correctly answered by more than half of the respondents (Table S3).

Variables that offered little or no differentiation or did not contribute to the underlying latent variable (f45, f47, f49, f52, f55 and f58) were not included in the IRT model. Question f59 had a negative discrimination and was therefore excluded as the probability of getting a correct answer decreased as respondent knowledge increased. Item f54 was the easiest for farmers to answer because it had the lowest difficulty coefficient of −2.42 and so only required a respondent with a relatively low level of knowledge trait to have a 50% chance of giving the correct the response (Table 1). Item f57 had the highest difficulty coefficient of −0.38 indicating that it was the most difficult of the included questions to answer correctly. However, all questions in the IRT model had difficulty coefficients < 0 indicating that they were generally easy and there were no questions which would separate respondents with average versus very high levels of knowledge. Item f56 had the highest discrimination coefficient of 1.99 indicating that it did a good job of differentiating between individuals with moderate‐to‐high knowledge and those with low knowledge.

**Table 1:** Item discrimination and difficulty coefficients of factors included in the item response theory model for ASF knowledge

Item name	Statement proposed to respondent	Discrimination		Difficulty	
		Coef.	*SE*	Coef.	*SE*
f46	Footbath at farm is a waste of money	1.23	0.11	−0.41	0.06
f48	My pigs can get sick when the traders get close to them	0.89	0.09	−1.34	0.13
f50	Birds or rodents can transmit the disease when they get in contact with the pigs	0.81	0.08	−0.76	0.09
f51	If I isolate the new pigs coming to my farm, I will stop the disease	0.76	0.09	−1.74	0.18
f53	If swill is heated before giving to pigs, chance of catching the disease is reduced	0.73	0.08	−0.49	0.09
f54	Burying dead pigs reduces the disease spread	0.88	0.11	−2.42	0.25
f56	Undisinfected farm tools can spread the disease	1.99	0.19	−0.73	0.05
f57	Use of disinfectant is not good for the pigs	1.81	0.17	−0.38	0.05

### 3.3 Knowledge gain by farmers following training

The variance component for village showed that there was some variation in response between the (randomly) selected villages and some clustering effect of respondents in the same village (ICC = 0.1), although it was small compared to the residual respondent‐to‐respondent variation (Table 2). District was a significant fixed effect (*p* < .001). Respondents initial starting level of knowledge also significantly, but negatively, influenced their change in knowledge (*p* < .001). There was significant effect of biosecurity training after 12 months (treatment), *p* = .038. Change in knowledge measured by the IRT latent variable showed a non‐significant average decrease of 0.09 (CI = −0.035, 0.213) in the control group, while the treatment group showed an average gain of 0.29 (CI = 0.167, 0.413). Production domain (peri‐urban/urban) and group membership positively influenced knowledge gain (both *p*‐values < .001).

**Table 2:** Random‐effects linear regression model of knowledge gain (between baseline and 12 months) with baseline knowledge as covariate (*significant at *p* < .05)

Factor (reference level in bracket)	Knowledge gain	
	Coef. (*SE*)	*p* > *t*
Trained (control)	0.18 (0.09)	.038*
Baseline knowledge	−0.70 (0.03)	.000*
Female (male)	0.03 (0.062)	.713
Lira urban (Lira rural)	−0.02 (0.12)	.880
Masaka rural	0.32 (0.12)	.006*
Masaka urban	0.52 (0.12)	.000*
Not belonging to farmer group (belonging)	−0.32 (0.07)	.000*
No formal education (primary or post‐primary)	0.04 (0.05)	.471
Constant	0.05 (0.12)	.661
Village variance	0.041 (0.02); CI95 (0.02; 0.09)	
Residual variance	0.54 (0.03); CI95 (0.49; 0.59)	

### 3.4 Impact of training and factors that influence farmer's attitude towards pigs and people's movements during ASF outbreaks

In general, veterinarians in the study region did not practise biosecurity measures before/after visiting pig farms. Despite this, over 84% of the respondents said that they would not stop veterinarians from entering their farms if there was an outbreak of ASF (f61) indicating an inadequate concern about veterinarian's personal biosecurity routine. Item f60 (stopping traders entering) had the highest proportion of respondents with positive attitude (70%) followed by f63 (not selling pigs during an outbreak) (52%) (Table S4).

Following training of farmers on biosecurity, there was no significant change on farmer's attitude towards restriction of farm visits by veterinarians and live pig traders and sale of pigs during outbreaks (Table 3).

**Table 3:** Random‐effects logistic regression analysis of factors that influence farmer's attitude towards pigs and human movements during ASF outbreaks (*significant at *p < *.05)

Factor (reference level in bracket)	I would stop traders from entering my farm if there is outbreak of ASF in my area (f60)		I would stop veterinarians from entering my farm if there is outbreak of ASF in my area (f61)		I would not sell my pig if there is outbreak of ASF in my area (f63)	
	Coef. (*SE*)	*p* > *t*	Coef. (*SE*)	*p* > *t*	Coef. (*SE*)	*p* > *t*
Trained (control)	0.17(0.17)	.34	−0.24(0.16)	.152	−0.05(0.120)	.625
Female (male)	−0.39(0.13)	.003*	−0.12(0.17)	.470	0.01(0.12)	.970
District domain (Lira rural)						
Lira urban	0.18(0.25)	.484	−0.02(0.24)	.943	−0.09(0.14)	.554
Masaka rural	0.32(0.23)	.166	0.77(0.23)	.001*	0.20(0.14)	.145
Masaka urban	0.59(0.25)	.018*	1.3(0.23)	.000*	0.14(0.15)	.325
Not belonging to farmer group (belonging)	−0.0001(0.16)	.995	−0.39(0.18)	.027*	0.02(0.14)	.895
Primary education and lower						
Education—none (primary or post‐primary)	0.39(0.13)	.002*	0.46(0.15)	.002*	−0.23(0.11)	.034*
Years in pig business	0.001(0.01)	.264	0.002(0.00)	.083	−0.01(0.00)	.002*
Constant	0.54(0.24)	.029*	−1.91(0.26)	.000*	−0.04(0.18)	.934
Village variance	0.38(0.08)		0.22(0.11)		0(0)	

#### 3.4.1 Deny traders access into their farm if there is an ASF outbreak

Gender, production domain and education were important factors that influenced farmer's attitudes towards denying traders access to their farms during ASF outbreaks. Female household heads were less likely to deny traders access to their farms than their male counterparts. Farmers from Masaka in both urban and rural areas were more likely to deny traders access to their farms than those in Lira.

#### 3.4.2 Allow veterinary officers access into their farm if there is an ASF outbreak

Production domain, education and belonging to farmer groups were important factors that influenced farmer's attitudes towards allowing veterinarians to access their farms during ASF outbreaks. Farmers in urban areas reported that they were likely to allow veterinarians to access their farms during outbreaks compared to farmers in rural Lira. Farmers who belong to groups were more likely to allow veterinarians into their farms compared to those who are not members of farmer groups.

#### 3.4.3 Sale of pigs during ASF outbreak

Education of the farmer and years of experience in pig production were the only important factors that influenced farmer's decision to sell the pigs when there was an outbreak of ASF in farms. Many educated farmers with more years of experience did not sell their pigs when there is an outbreak of ASF in their farms.

### 3.5 Impact of training and factors that influence biosecurity practices

Items (f66, f68, f72, f74, f76 and f77) were analysed. From Table S5, the distribution patterns for practice are similar for both the control and trained groups, reflecting lack of change after training.

Training on biosecurity had a positive influence on home slaughter. Location (Masaka urban) and education (informal education) had a positive influence on isolation of newly acquired pigs. Location (Masaka rural) and not belonging to a farmers group had negative influence in use of disinfectant, while location (Lira urban) and informal education positively influenced use of disinfectants. Processing of swill prior feeding to pigs was negatively influenced by location (rural) and positively influenced by experience in the pig business. Women farmers were more likely to deny entry to visitors other than the family members on the farms, while location (Masaka urban and rural) and experience in pig business have a positive influence on restriction of farm visits by traders (Table 4).

**Table 4:** Factors that influence change in practices related to ASF biosecurity (*significant at *p < *.05)

Factor (reference level in bracket)	Do you slaughter pigs at home (f66)		Do you isolate newly purchased stock before mixing with the existing stock (f68)		Do you use disinfectants on farm (f72)		Do you feed your pigs on un‐processed swill (f74)		I allow people other than my family members to enter my pig pen (f76)		I allow traders to enter my pig pen (f77)	
	Coef. (*SE*)	*p* > *t*	Coef. (*SE*)	*p* > *t*	Coef. (*SE*)	*p* > *t*	Coef. (*SE*)	*p* > *t*	Coef. (*SE*)	*p* > *t*	Coef. (*SE*)	*p* > *t*
Trained (control)	0.009(0.21)	.040*	0.041(0.14)	.757	−0.139(0.20)	.476	−0.115(0.17)	.504	−0.013(0.14)	.916	0.109(0.12)	.362
Female (male)	0.016(0.15)	.110	0.036(0.13)	.777	0.097(0.18)	.587	−0.201(0.13)	.125	−0.266(0.122)	.030*	−0.177(0.12)	.156
District domain (Lira rural)												
Lira urban	−0.055(0.301)	.857	−0.048(0.19)	.0806	0.507(0.25)	.047*	−0.410(0.25)	.096	0.272(0.192)	.157	0.264(0.17)	.132
Masaka rural	0.214(0.289)	.459	0.433(0.18)	.017*	−1.529(0.31)	.000*	1.111(0.23)	.000*	−0.077(0.18)	.665	−0.574(0.16)	.000*
Masaka urban	0.235(0.299)	.431	0.373(0.19)	.052	−0.46(0.27)	.082	−0.469(0.24)	.049*	0.088(0.19)	.635	−0.766(0.17)	.000*
Not belonging to farmer group (belonging)	0.016(0.186)	.931	−0.124(0.15)	.423	−0.968(0.20)	.000*	−0.003(0.16)	.829	0.084(0.15)	.567	0.030(0.15)	.840
No formal education^a^(primary or post‐primary)	0.131(0.139)	.777	0.416(0.12)	.000*	0.773(0.16)	.000*	0.178(0.12)	.133	−0.001(0.113)	.994	0.010(0.11)	.933
Years in the pig business	−0.000(0.00)	.345	−0.002(0.00)	.122	−0.001(0.01)	.760	−0.004(0.001)	.003*	0.000(0.00)	.769	−0.003(0.001)	.021*
Constant	1.189(0.291)	.000*	−0.093(0.21)	.662	−1.083(0.29)	.000*	−0.313(0.24)	.188	0.229(0.20)	.257	0.294(0.19)	.129
Village variance	0.487(0.099)		0.231(0.08)		0.326(0.11)		0.377(0.08)		0.231(0.08)		0.160(0.09)	

^a^ No formal education is referred as short‐ or long‐term trainings given to pig producers usually by government extension services or development organizations through projects.

### 3.6 Perception of farmers about implementation of biosecurity measures

During the FGDs, reasons for low adoption of biosecurity practices were discussed. High financial cost of practices and community stigmatization were among the major issues highlighted (Table 5).

**Table 5:** Main reasons for failing to apply important biosecurity measures according to farmers (from focus group discussions)

Measures that were difficult to implement by farmers	Reasons given for not implementing the measures
Construction of fences/pig structures/housing	High financial cost Lack of knowledge on design of appropriate pig house
Limiting visitors from going to the pig units	Community stigma No means for estimating pig weight at selling
Disposing of dead pigs by burying	Lack of land to bury carcasses; their piece of land is either small or rented. Some communities consume the dead pigs Requires labour
Disposing of dead pigs by burning	High financial cost (requires fuel) Safety issues (fear of bush fire) Environmental pollution (because of the smoke)
Stopping the use of communal boars for breeding	Expensive to own and raise a boar Sociocultural barriers for keeping a boar (for those with children, they fear would make them learn bad manners when they see a boar mounting a sow)
Use of disinfectant and footbath at the farm	Expensive and not feasible for all types of keeping Sociocultural barriers (fear that it may stop people from visiting them)
Boiling swill prior feeding pigs	High financial cost (requires wood)
Isolating sick pigs from healthy ones	Farmers have small plots of land, causing limited space for extra room for pig house
Keeping away animals from the farm such as dogs and other pigs	Difficult to achieve when pigs are scavenging or tethered
Informing authorities about an ASF outbreak in an area	Limited access of farmers to veterinary authorities Slow and limited actions taken by authorities when informed about suspected outbreaks

## 4 DISCUSSION

Implementation of biosecurity is key to successful pig production in an ASF‐endemic environment (Fasina, Lazarus, Spencer, Makinde, & Bastos, 2012). However, knowledge of biosecurity's key principles is fundamental if farmers want to substantially change their perception of disease risks and consequently increase their level of awareness of the importance of biosecurity measures. Several studies recommend training of pig farmers on strict biosecurity measures as a means of mitigating ASF in Uganda (Dione et al., 2014, 2015; Kabuuka et al., 2014). Increased understanding of pig production and specifically biosecurity and disease control practices was also considered by Vietnamese pig farmers as important to improve productivity (Barot, 2017). Past studies pointed out the lack of knowledge of Ugandan pig farmers as a key challenge to pig management including diseases (Dione, Ochago, et al., 2016; Dione, Ouma, et al., 2016; Dione et al., 2014; Ouma et al., 2015). This is what prompted our research which aims at evaluating the effect of training farmers on best practices of biosecurity. In Uganda, training has been offered to pig farmers by local government extension personnel, non‐governmental organizations and private veterinarians on topics such as pig feeding, health and farm management (ILRI, 2013). However, most of the training is sporadic, one‐off and mapped to specific development projects, with no formal evaluation of its impacts on the pig farms locally and nationally. Our study proved that training can significantly improve farmer's knowledge of biosecurity. However, different explanatory factors seem to be influencing different biosecurity items. This shows the high variability of the smallholder pig production systems, and the complexity of biosecurity, which make it difficult to grasp the key factors that influence biosecurity practices. It was shown in our study that experience in the pig business and group membership have positive influence on adoption of good biosecurity practices by farmers. In fact, in the study areas, pig farmers were encouraged to form groups to apply collective marketing and get access to training. In Nigeria, it was also shown that farmer's group have a high impact in adoption of technological innovations (Kolade & Harpham, 2014). Therefore, farmer groups should be supported and could constitute an entry point for biosecurity trainings. However, limited change of farmers in attitude and practices towards biosecurity after several months of follow‐up was noticed. Our finding supports the conclusion of a study in Northern Uganda by Chenais et al. (2015) that lack of knowledge may not be the major driver of the continuous circulation of ASF virus in this setting, since farmers showed willingness to learn and capacity to internalize knowledge. Several studies in livestock systems have reported a range of factors that limit adoption of biosecurity measures by farmers in different livestock production contexts. These include limited available resources and access to knowledge, local context, nature of the target disease, sociocultural and religious practices (Gunn, Heffernan, Hall, McLeod, & Hovi, 2008; Sayers et al., 2013; Toma, Stott, Heffernan, Ringrose, & Gunn, 2013; Young et al., 2015). In a specific smallholder pig sector such as Nigeria, according to Fasina et al. (2012) additional workforce, costs and complexities of applications, availability of funds, laws, and regulations affecting the producers’ decision about a new biosecurity plans are key inhibitors of adoption. According to Can and Altuğ (2014), there were statistically significant associations between the producers’ socio‐economic characteristics and some of the biosecurity practices. As supported by Ouma et al. (2018) in our study area, we think that greater importance should be given to financial support to farmers through market‐based model, as well as exploration of cheaper options to available biosecurity measures that lower financial cost barrier practices. For instance, the use of disinfectants made using locally available ingredients may be an economical alternative to commercial products.

Live pig traders and pork butchers were pointed out as a major risk for the spread of ASF between farms in Uganda (Dione, Ochago, et al., 2016; Dione, Ouma, et al., 2016). Our study found that farmer's attitude towards restriction of trader and veterinarian's movements to the farm during disease ASF outbreaks did not significantly change after the training. This could be related to the stigma associated with restricting movement that farmers said was a key issue in their communities. However, women were less likely to allow veterinarians without protective clothing into their farms, but more likely to allow other type of visitors such as neighbours and traders to enter they farms. This could be explained by the fact that the contact between married women in the absence of their husband and educated men (as it is the case with the veterinarians) is not acceptable in this society. Another explanation could be that in male‐headed households, women usually do not hold the money to pay to the veterinarians. If men are the ones who pay bills, so in their absence, women cannot take a decision to engage veterinarians. But when it comes to selling their animals, traders are the only option, so they are generally welcome without restriction. In Uganda, women perform most of the pig management activities; hence, they play a critical role in pig husbandry and biosecurity (Dione, Ochago, et al., 2016; Dione, Ouma, et al., 2016; Ouma, Ochago, Dione, Birungi, & Lule, 2016). Therefore, there is need to consolidate biosecurity training and deliberately target them. According to Young, Suon, Andrews, Henry, and Windsor (2013), behaviour change towards adopting improved biosecurity is likely to have positive benefits and impacts on the smallholder livelihood and greater public good. However, positively influencing the development of the smallholder farming system through ‘uptake and adoption’ of sustainable interventions or ‘change’ is a major challenge, particularly with respect to improving the management of disease risks (Young et al., 2015). Keys actions for behavioural change include better coordination between stakeholders to encourage a shared biosecurity understanding and improve awareness of the importance of biosecurity practices within the broader animal health system (Hernandez‐Jover, Higgins, Bryant, Rast, & McShane, 2016). Profitability remains the principal driver for involvement in pig rearing; hence, the understanding of this factor and its use in the introduction and maintenance of principles of biosecurity at farm level becomes important for controlling ASF in small‐ to medium‐scale piggeries and farming communities (Fasina et al., 2012). In the Uganda pig systems, numerous other factors that negatively affect pig performance, especially lack of good quality feeds and limited access to market (ILRI, 2013), should be tackled at the same time as ASF.

In the overall context of the smallholder livestock systems, where farmers are faced with a variety of challenges, adoption and behavioural change regarding biosecurity are multifaceted and need to be addressed using an integrated package considering farmers perception of disease risk, their motivations and abilities to make informed decisions based on what they consider as a priority in relation to the pig business. Kabuuka et al. (2014) suggested that training for small‐scale and emerging pig farmers in Uganda should involve multidimensional and multidisciplinary approaches to reduce human‐related risky behaviour driving infection. Therefore, implementation of biosecurity could benefit from a one health approach by looking at biosecurity measures in a more holistic way, which would probably make training more attractive to farmers since it will also be tackling issues that directly affect farmer's own health. Results of this study pointed towards potential important factors that should be further considered when implementing biosecurity training.

Limitations of this study include possible spillover of information given that it was impossible to control information sharing among farmers between villages. All interviewers were sourced from the district veterinary office; hence, they are very socially close to the farmers. Therefore, bias associated with the nature of interviewers must also be considered. On some occasions, some farmers might have gaven misleading responses to hide their true perceptions. Pig‐keeping households were identified through a census by the District Veterinary Office, hence there is a risk of not having an exhaustive list of farmers.

## 5 CONCLUSION

Participatory training improved knowledge of biosecurity. However, limited adoption of biosecurity practices and change in attitude of farmers towards implementation of biosecurity were noticed. Gender, production domain, group membership and education were important factors that influenced knowledge gain, attitude and practices of farmers towards biosecurity. This paper provides empirical evidence on the impact of training intervention on biosecurity practices for disease prevention or control. In addition, it breaks down the components of the biosecurity practices and documents the specific challenges to its uptake by the farmers. It therefore relaxes the assumption of knowledge constraint as a barrier to uptake. The results clearly show that knowledge is not the binding constraint to uptake of the biosecurity interventions.

## CONFLICT OF INTEREST

The authors declare that there are no conflicts of interest.

## Supporting information

Sup info

## Data Availability

The data that support the findings of this study are available from the corresponding author upon reasonable request.
