# Supplementary material for: Impact of participatory training of smallholder pig farmers on knowledge, attitudes and practices regarding biosecurity for the control of African swine fever in Uganda
Source: Transbound Emerg Dis. 2020 May 17;67(6):2482–93. doi: 10.1111/tbed.13587 (PMC7754142; doi:10.1111/tbed.13587)
Supplement: Supplementary file 1 — Sup info [file TBED-67-2482-s001.docx]

**Appendices**

Supplementary 0: Diagram of the study design showing the different steps and activities carried out during the study

Study design, site selection and logistical set-up

KAP on ASF disease and biosecurity

Baseline data collection (April 2015)

Intervention (training of farmers in treatment group)

Refresher training after three months (treatment group)

Data collection at 12 months after baseline (May 2016)

KAP on biosecurity

Data collection at 28 months after baseline (September 2017)

Treatment group only - KAP on biosecurity

Focus group discussions

End of study (training of farmers in the control groups)

Supplementary 1 : Information components of Knowledge Attitude and Practice questionnaires (questionnaire available in supplemental material)

| Biosecurity | Type of questions | | | Question reference |
| --- | --- | --- | --- | --- |
| Topic | Knowledge  (Likert-scale) | Attitude  (Likert-scale) | Practice (open-ended) |  |
| Pig keeping type (free range or confined) |  |  | x | f26 |
| Pig housing/farm fencing | x |  | x | f45, F47 |
| Trader entry to farm | x | x | x | f48, f77 |
| Veterinarian entry to farm | x | x |  | f49 |
| Neighbour, relative visit to farm | x |  | x | f62, f76 |
| Bird and rodent control | x |  |  | f50 |
| Isolation of new stock | x |  | x | f51, f68 |
| Farm cleaning | x |  | x | f52, f69 |
| Farm disinfection | x |  |  | f57, f72 |
| Swill processing | x |  |  | f52, f74 |
| Management of dead pigs | x |  |  | f54 |
| Management of offal after slaughter | x |  |  | f55 |
| Disinfection of farm tools | x |  | x | f56, f77 |
| Management of stray dogs | x |  |  | f58 |
| Consumption of meat from pigs with ASF | x |  |  | f59 |
| Pig confinement during outbreak |  | x |  | f64 |
| Sale of sick pigs during outbreak |  | x |  | f63 |
| Footbath at farm entry | x |  | x | f46, f70 |
| Home slaughter |  |  | x | f66 |
| Pig transportation |  |  | x | f65 |

Supplementary 2: Proportion of household demographic characteristics

| Characteristics | Variable | Trained (%) | Untrained (%) |
| --- | --- | --- | --- |
| Gender of household head | Male | 79.7 | 72.7 |
|  | Female | 20.3 | 27.3 |
| Gender of respondent | Male | 44.1 | 39.9 |
|  | Female | 55.9 | 60.1 |
| Highest education level | No formal education | 9.5 | 8.9 |
|  | Primary | 49.4 | 58.4 |
|  | Secondary | 28.8 | 20.8 |
|  | Tertiary/university | 12.3 | 11.9 |
| Production domain | Rural | 58.6 | 56.5 |
|  | Urban | 41.4 | 43.5 |
| Respondent role in the farm | Management | 98.1 | 97.5 |
|  | Marketing & other | 1.9 | 2.5 |
| Pig keeping practices | Housed | 62.0 | 40.0 |
|  | Tethered | 35.0 | 55.0 |
|  | Free range | 3.0 | 5.0 |
| Pig feeding practices | Rainy season | | |
|  | Crop residues | 89.0 | 81.0 |
|  | Forages | 5.1 | 9.4 |
|  | Concentrates | 5.7 | 9.4 |
|  | Swill feeding | 0.0 | 0.2 |
|  | Scavenging | 0.2 | 0.0 |
|  | Dry season | | |
|  | Crop residues | 67.5 | 56.4 |
|  | Forages | 7.2 | 7.7 |
|  | Concentrates | 19.6 | 28.2 |
|  | Swill feeding | 4.1 | 7.7 |
|  | Scavenging | 1.7 | 0.0 |
| Number of respondents | | 425 | 405 |

Supplementary 3: Knowledge item description and proportion of individuals responding to each question with a desirable response (good knowledge) over both baseline and 12-month surveys.

| Item name | Statement proposed to respondent | Baseline (%) | | 2nd phase (%) | |
| --- | --- | --- | --- | --- | --- |
|  |  | Trained | Not trained | Trained | Not trained |
| f45* | Housed pigs will not catch ASF | 46.6 | 53.4 | 46.4 | 53.6 |
| f46 | Footbath at farm is a waste of money | 50.8 | 49.2 | 56.0 | 44 |
| f47* | If I fence my house, the pigs will not catch the disease | 48.0 | 52 | 49.9 | 50.1 |
| f48 | My pigs can get sick when the traders get close to them | 52.2 | 47.8 | 54.2 | 45.8 |
| f49* | My pigs can get sick when the veterinarians get close to them | 52.0 | 48 | 59.3 | 40.7 |
| f50 | Birds or rodents can transmit the disease when they get in contact with the pigs | 52.8 | 47.2 | 53.9 | 46.1 |
| f51 | If I isolate the new pigs coming to my farm, I will stop the disease | 51.3 | 48.7 | 52.8 | 47.2 |
| f52* | Pigs will catch the disease if the farm is clean | 48.9 | 51.1 | 52.7 | 47.3 |
| f53 | If swill is heated before giving to pigs, chance of catching the disease is reduced | 49.4 | 50.6 | 55.0 | 45 |
| f54 | Burying dead pigs reduces the disease spread | 51.8 | 48.2 | 52.7 | 47.3 |
| f55* | Pigs will not get sick when they ingest offal from infected dead pigs | 49.5 | 50.5 | 51.1 | 48.9 |
| f56 | Undisinfected farm tools can spread the disease | 50.8 | 49.2 | 53.3 | 46.7 |
| f57 | Use of disinfectant is not good for the pigs | 48.7 | 51.3 | 54.3 | 45.7 |
| f58* | I should avoid stray dogs from coming close to my pigs because they can transmit the disease to them | 50.9 | 49.1 | 53.8 | 46.2 |
| f59** | The meat of dead pigs of ASF is safe for human consumption | 61.0 | 39.0 | 47.8 | 52.2 |

* excluded in the IRT model because they offered little or no differentiation, or did not contribute to the underlying latent variable

** excluded in the IRT model because it had a negative discrimination

Supplementary 4: Attitude item description of individuals with a desirable response (good attitude)

| Item name | Statement proposed to respondent | Baseline (%) | | 2nd phase (%) | | 3rd phase (%) |
| --- | --- | --- | --- | --- | --- | --- |
|  |  | Trained | Not trained | Trained | Not trained | Trained |
| f60 | I would stop traders from entering my farm if there is outbreak of ASF in my area | 71.8 | 70.7 | 72.2 | 63.3 | 70.7 |
| f61* | I would stop veterinarians from entering my farm if there is outbreak of ASF in my area | 17.2 | 17.4 | 12.8 | 15.4 | 15.6 |
| f63 | I would not sell my pig if there is outbreak of ASF in my area | 42.7 | 52.0 | 59.0 | 52.9 | 47.3 |

*veterinarians are known to move from farm to farm without protective wears and biosecurity measures.

Supplementary 5: Percentage of households practicing recommended animal biosecurity practices (good practice) by treatment group

| Item name | Statement proposed to respondent | Baseline (%) | | 2^nd^ phase (%) | | 3^rd^ phase (%) |
| --- | --- | --- | --- | --- | --- | --- |
|  |  | Trained | Not trained | Trained | Not trained | Trained |
| f66 | Do you slaughter pigs at home | 80.2 | 77.8 | 78.2 | 78.1 | 18.2 |
| f68 | Do you isolate newly purchased stock before mixing with the existing stock | 53.2 | 55.8 | 60.8 | 54.1 | 69.1 |
| f72 | Do you use disinfectants on farm | 13.3 | 16.0 | 15.8 | 14.0 | 12.7 |
| f74 | Do you feed your pigs on un-processed swill | 46.0 | 41.2 | 46.7 | 52.9 | 48.0 |
| f76 | I allow people other than my family members to enter my pig pen | 56.3 | 58.9 | 56.6 | 54.8 | 78.9 |
| f77 | I allow traders to enter my pig pen | 52.2 | 52.5 | 57.8 | 53.1 | 72.3 |
